# County-Level Factors That Influenced the Trajectory of COVID-19 Incidence in the New York City Area

**DOI:** 10.1089/hs.2020.0236

**Published:** 2021-06-17

**Authors:** Ashley Wendell Kranjac, Dinko Kranjac

**Affiliations:** Ashley Wendell Kranjac, PhD, is an Assistant Professor, Department of Sociology, Chapman University, Orange, CA. Dinko Kranjac, PhD, is an Assistant Professor, Department of Psychology, University of La Verne, La Verne, CA.

**Keywords:** COVID-19, SARS-CoV-2, Community health, Public health preparedness/response, Epidemic management/response

## Abstract

More than a century of research has shown that sociodemographic conditions affect infectious disease transmission. In the late spring and early summer of 2020, reports of the effects of sociodemographic variables on the spread of COVID-19 were used in the media with minimal scientific proof attached. With new cases of COVID-19 surging in the United States at that time, it became essential to better understand how the spread of COVID-19 was varying across all segments of the population. We used hierarchical exponential growth curve modeling techniques to examine whether community socioeconomic characteristics uniquely influence the incidence of reported COVID-19 cases in the urban built environment. We show that as of July 19, 2020, confirmed coronavirus infections in New York City and surrounding areas—one of the early epicenters of the COVID-19 pandemic in the United States—were concentrated along demographic and socioeconomic lines. Furthermore, our data provides evidence that after the onset of the pandemic, timely enactment of physical distancing measures such as school closures was essential to limiting the extent of the coronavirus spread in the population. We conclude that in a pandemic, public health authorities must impose physical distancing measures early on as well as consider community-level factors that associate with a greater risk of viral transmission.

## Introduction

The presence of SARS-CoV-2 was initially detected in Wuhan, China, in December 2019.^1^ Since then, the outbreak of COVID-19, the disease caused by SARS-CoV-2, has spread worldwide.^2^ As of July 19, 2020, the United States found itself atop the list of countries most heavily impacted by the pandemic, in terms of both total diagnoses and total fatalities.^3^ Within the United States, the residents of the state of New York were, until mid-May of 2020, disproportionately affected by COVID-19, with New York City accounting for the vast majority of cases and deaths in the country.^4^

Following the first confirmed New York case in March 2020, the incidence of COVID-19 in and around New York City followed an alarming exponential rise.^4^ This upward trend, however, was not uniformly patterned, as evidenced by the substantial spatial heterogeneity of confirmed cases.^5^ Larger population size increases the potential for virus transmission and may, in part, account for geographic differences in numbers of COVID-19 cases.^6^ However, in addition to the readily predictable effects of population concentration, communities also exhibited clear stratification along dimensions of demographic and socioeconomic characteristics, such as poverty, that associate with vulnerability and, in turn, influence disease patterning.^7^

Relevant to our study, published reports, such as those discussed by Quinn and Kumar,^8^ indicate that social conditions and community-level characteristics affect the incidence of numerous communicable diseases. Indeed, social epidemiologists and medical sociologists argue that individuals and groups possessing fewer social, educational, and economic resources may prove less able to avoid areas where infectious disease runs rampant.^6-8^ Notably, counties in the New York City area are some of the most racially and ethnically diverse in the world.^9^ The population of Queens County in 2019, for example, was 26.9% Asian, 20.7% Black, 28.2% Hispanic, and 24.2% White.^9^ Moreover, levels of poverty also vary substantially across counties in the New York City area.^9^ In 2019, for example, 27.3% of people residing in Bronx County, compared with 5.8% of those in Nassau County, lived in poverty.^9^ In the current study, we proposed that differences in existing demographic, socioeconomic, and structural variables may have contributed to community-level differences in COVID-19 infection rates in the New York City area.

Here, we used an index of concentrated disadvantage as a socioeconomic indicator of the individual's county of residence. Concentrated disadvantage is a multidimensional population-level measure that is typically constructed using several components of deprivation, such as mean levels of educational attainment, levels of poverty, rates of unemployment, racial/ethnic composition, percentage of female-headed households, and percentage of individuals receiving public assistance.^10^ It is important to note that measures of concentrated disadvantage are known to independently associate with disease patterning, including infectious disease transmission,^11^ but data specific to COVID-19 outbreaks are inconsistent.^11-15^ More specifically, findings vary substantially by the modeling technique used, inclusion or exclusion of independent variables, granularity (eg, county level vs state level), and domain of the outcome being measured (eg, concentrated disadvantage vs social vulnerability index).^11-15^ Our study adds to the existing literature and helps clarify the impact of county-level demographic and socioeconomic disparities on COVID-19 incidence in the New York City area.

## Methods

Our primary outcome of interest was the spread of COVID-19 across 7 counties in the New York City area. We extracted data on the first recorded case in each county, followed by the number of daily confirmed cases per county, from USAFacts, which confirms county-level data by directly referencing both state (New York State Department of Health) and local (New York City Department of Health and Mental Hygiene) public health agencies.^16^ We measured time as the number of days since the first diagnosed case per county, and included K-12 school closure dates in the analysis to account for county-by-county variations in time of enactment of physical distancing measures since the first reported case.^17,18^ We included a spatial lag composed of an inverse-distance spatial weighting matrix to account for dependencies in counties that are geographically adjacent. We used Stata version 16 (StataCorp, College Station, TX) for our analyses.

County-level measures of socioeconomic factors were generated using data from the 2014-2018 American Community Surveys.^9^ Measures included total population size, as an offset to denote the size of the population at risk; median age; and percentages of adults with less than 12 years of education, Black residents, individuals below the federal poverty level, individuals receiving public assistance, female-headed households, and the population unemployed. We followed Sampson et al^10^ to generate a standardized index variable of concentrated disadvantage. We used the first loading of a principal components factor analysis on all the county-level characteristics described, with the exception of population size and median age, to generate an overall *z*-score transformed index ranging from lowest disadvantage (*z*-score = -1.00) to highest disadvantage (*z*-score = 2.09). The eigenvalue of this index was greater than 1, and internal consistency, as measured by Cronbach alpha, was high at 0.92 (Table 1). We then used this index of concentrated disadvantage along with median age and a measure of population size as county-level socioeconomic indicators.

**Table 1.** Results From the First Loading of a Principal Components Factor Analysis Across 7 New York Counties

Concentrated Disadvantage	
Variable	Factor
% adults <12 years education	0.35
% Black residents	0.13
% below poverty line	0.14
% on public assistance	0.10
% female-headed families	0.19
% unemployed	0.12
Eigenvalue	6.64

Data are from the United States Census Bureau.^9^

Our aim was to uncover whether the rate of viral transmission varied by the index of concentrated disadvantage once the virus entered a county. Because we were analyzing relatively rare events within small units, we tested the impact of distinct sociodemographic environments on COVID-19 counts using hierarchical Poisson exponential growth curve modeling techniques that could account for overdispersion and correlation in the number of cases by county. All models used maximum likelihood estimation with adaptive quadrature that adjusted for problems that would otherwise downwardly bias the estimated standard errors.^19^

A prior report,^20^ as well as our exploratory analyses, indicate that due to nonlinearity, time is most appropriately captured by a cubic time function, and thus we present only results from these models. We specify that these counts have variable exposure by county population and thereby make the analysis one of COVID-19 rates.^21^ Since COVID-19 infection occurs at most once for each confirmed case, and population total is included as an offset to denote the size of the population at risk of infection, we refer to the coefficients in our models as incidence rate ratios (IRRs).^21^ In the models, time points are nested within counties. The fully specified level 1 model fits the number of cases per day as a function of time across observations for each county, while simultaneously accounting for variation in the enactment of the physical distancing measures and a spatial lag. The fully specified level 2 model fits the level 1 intercept and coefficients across all counties as a function of the county-level characteristics. All models treat the intercept as random across counties.

## Results

Supplementary Table 1 (www.liebertpub.com/doi/suppl/10.1089/hs.2020.0236) displays the number of confirmed COVID-19 cases and the estimated means and standard deviations of the covariates for each county. As of July 19, 2020, Queens County had the highest number of reported cases (n = 67,007), followed by Kings County (n = 61,432), whereas New York County had the lowest number of reported cases (n = 29,731). Enactment of K-12 school closures across the state began on March 16, 2020, which was 9 days after the first reported case in Bronx and Suffolk counties, but 15 days after the first reported case in New York County. The median age of the population varied significantly across counties, from a high of 42 years in Nassau County to a low of 34 years in Bronx County. Population size also varied across counties, with Kings County being the most populated (2,559,903). Relative to other counties, Bronx County and Kings County were the most disadvantaged, with the highest percentages of the population below poverty (26.1% and 17.1%, respectively), the highest unemployment rates (6.3% and 4.4%), and the highest percentages of the population receiving public assistance (7.9% and 4.8%). On the other end of the spectrum, Nassau County was the least disadvantaged relative to other counties, with the lowest percentage of the population below poverty (4.0%), the lowest unemployment rate (2.8%), and the lowest percentage of the population receiving public assistance (1.3%).

Because we were interested in trajectories of viral transmission once the virus entered a county, we used the first recorded case in each county as the baseline of that trajectory. We then estimated the growth patterns of COVID-19 incidence across counties over time, with the spatial lag and physical distancing measures included at model level 1 and the county-level characteristics of median age and concentrated disadvantage included at model level 2. The results from our hierarchical Poisson cubic growth curve models are shown in Table 2. The exponentiated regression coefficients are displayed in terms of IRR and represent the relative change in the rate of infection per standardized population size over time. We controlled for population size in all models.

**Table 2.** Incidence Rate Ratios for Hierarchical Poisson Polynomial Growth Regression Models Predicting Confirmed COVID-19 Cases (N = 327,578)

	Full Model		
Fixed Effects	IRR	*P* Value	95% CI
Time	1.23	<.001	1.23–1.23
Time^2^	1.00	<.001	1.00–1.00
Time^3^	1.00	<.001	1.00–1.00
Physical distancing			
K-12 school closure	0.10	<.001	0.09–0.12
Spatial lag	1.00	<.001	1.00–1.00
Median age	1.13	<.001	1.08–1.17
Population total	1.00	<.001	1.00–1.00
Concentrated disadvantage (SD)	1.29	<.01	1.16–1.49
*Variance of Random Effect*	*b*		*95% CI*
	0.02	<.001	0.01–0.03

Data are from March 2 through July 19, 2020, and are drawn from USAFacts,^16^ The City of New York website,^5^ Suffolk County Government,^18^ and the United States Census Bureau.^9^

^a^ Time is measured as the number of days since the first diagnosed case per county. Prior reports,^20^ as well as our exploratory analyses, indicate that time is most appropriately captured by a cubic time function due to nonlinearity.

Abbreviations: CI, confidence interval; IRR, incidence rate ratio; SD, standard deviation.

As shown in Table 2, from the onset of the outbreak, without the physical distancing measures enacted, and when holding population size and spatial differences constant, each standard deviation increase in the linear (IRR 1.23, *P* < .001), quadratic (IRR 1.00, *P* < .001), and cubic (IRR 1.00, *P* < .001) components of the time slope associate with a relative increase in the IRR of COVID-19 infection. The model indicates further that the enactment of physical distancing control efforts associates with a relative reduction in the rate of reported cases (IRR 0.10, *P* < .001). Turning next to the county-level factors, every 1 standard deviation increase in median age across counties is associated with a relative increase in the IRR of COVID-19 infection (IRR 1.13, *P* < .001). After accounting for all the county-level characteristics, and with spatial and population size differences held constant, each standard deviation increase in concentrated disadvantage associates with a significant relative increase in the rate of COVID-19 incidence (IRR 1.29, *P* < .001). As shown in the lower portion of Table 2, there is significant variation between counties in initial COVID-19 incidence (*b* 0.02, *P* < .001).

**Figure 1.**
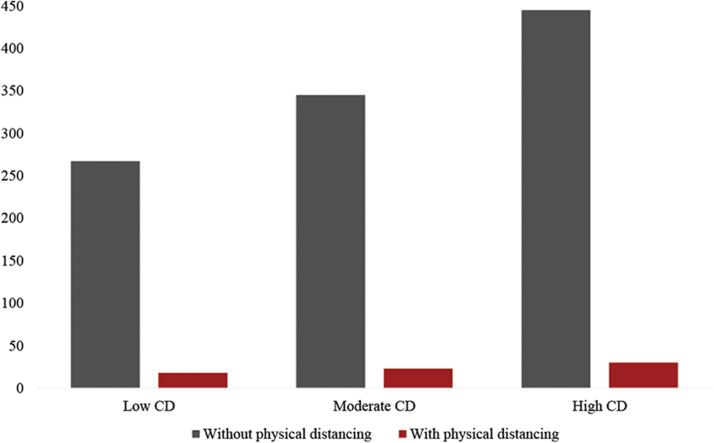
Fully adjusted predicted probabilities of COVID-19 infection. We trichotomized our standardized index of concentrated disadvantage into equal thirds, with and without physical distancing measures enacted, to estimate the predicted probabilities of COVID-19 incidence for counties. Abbreviation: CD, concentrated disadvantage.

To illustrate the patterns of COVID-19 incidence within these contexts, we divided the counties' levels of concentrated disadvantage into equal thirds and presented the predicted probabilities of COVID-19 incidence for the counties in each level, with and without physical distancing measures enacted, in Figure 1. Within communities facing the highest levels of concentrated disadvantage, we see a significantly higher probability of COVID-19 infection relative to the probability of infection among those living in areas of low levels of disadvantage (445.62 vs 267.68, *P* < .001), even with physical distancing measures enacted (30.13 vs 18.10, *P* < .001). Thus, our results present further evidence that concentrated disadvantage influences COVID-19 infection rates differently, with the most disadvantaged areas largely suffering the highest number of cases, both before and after the enactment of physical distancing measures.

## Discussion

Overall, our results, based on data collected from March 2 through July 19, 2020, indicate that the number of documented COVID-19 infections varies widely both *between* counties and over time *within* counties. For all counties, we observed a substantial increase in the daily number of diagnoses along with evidence that earlier enactment of physical distancing control policies, here measured by K-12 school closure dates,^17,18^ likely altered the trajectory of the outbreak. This latter finding clearly underscores the importance of timely introduction of physical distancing measures to prevent virus diffusion, particularly when used in combination and enacted without delay, as reported elsewhere.^22^

Even with physical distancing intervention efforts in place, people living in areas characterized by high concentrations of disadvantage are at greater risk of COVID-19 infection. This finding is in line with prior reports: although the enactment of physical distancing policies may help mitigate early spread of COVID-19 in urban areas with large populations,^23^ this benefit is not uniformly distributed across counties. To minimize viral transmission, public health officials must institute a number of well-timed physical distancing interventions, including closures of schools and workplaces, restrictions on mass gatherings and cancellations of large-scale public events, and restrictions on movement such as stay-at-home orders.^6,22,23^ However, we emphasize the fact that residents from poor communities often live in multigenerational homes and perform jobs that cannot be done remotely, and are thus less able to physically isolate and socially distance, which will shape the spread of infection and influence health outcomes.^24,25^ Consequently, persons from low-resource communities, compared with those living in more affluent areas, may benefit less from these mandated orders.^24^ Indeed, employees in some occupations (eg, healthcare, social assistance, agriculture, construction, manufacturing) have been shown to be at higher risk of COVID-19 exposure, which has major implications considering that racial/ethnic minority populations are overrepresented in these industry sectors.^24,25^ Related, transmission of COVID-19 often occurs within the household,^26-28^ and average US household size differs considerably by race/ethnicity.^29^ Public health programs seeking to eliminate or reduce inequities in viral transmission need to bolster testing uptake among the most vulnerable populations disproportionally affected by COVID-19.^30^

For well over a century, research has shown that many sociodemographic conditions affect infectious disease transmission, especially in urban settings.^6-8^ Here, we add to this literature and show that concentrated disadvantage is a potent ingredient in the observed pattern of detected COVID-19 infections across counties in the New York City area. We see this as evidence that the influence of communities on the spread of infectious disease reaches far beyond the physical characteristics of the urban built environment.^31^ Furthermore, if community sociodemographic data exhibit elevations in median age or higher prevalence of disadvantage (eg, poverty), such factors will likely have far-reaching consequences for COVID-19 incidence, as well as COVID-19-related case fatality rates.^32^

Although it is beyond the scope of our analysis to explain precisely why this variation exists, COVID-19 has exposed major social and economic disparities that persist in the United States. Indeed, individuals' risk of being infected is higher if they reside in counties of New York with more socioeconomic disadvantage.^6,12,15^ It is clear that social inequalities brought on by institutional and cultural racism in the United States (eg, historical and contemporary residential segregation policies) are key to understanding the persistence of health inequalities (eg, higher rates of disease, greater severity of illness, poorer survival rates) among racial/ethnic minorities.^33^ Because marginalized populations are underserved and particularly vulnerable to the effects of the pandemic,^8,11-15^ government officials must build a more robust public health system that will enable a more equitable response to the emerging health crisis.^34^ These are urgent matters that need to be addressed through multifaceted, evidence-based, and population-wide initiatives aimed concomitantly at poverty and racism to improve health and reduce disparities in health.

To the best of the authors' knowledge, this is the first study that employs hierarchical exponential growth curve modeling techniques to examine whether county-level demographic and socioeconomic characteristics influence the incidence of COVID-19 cases across counties in the New York City area. This study, however, is not without limitations. The available reports indicate that, especially during the early phase of the COVID-19 outbreak, limited diagnostic testing or hospital bed capacity, as well as testing or reporting delays, may influence the number of daily confirmed cases across counties.^20^ However, in New York City, the case incidence curve paralleled the growth rate of hospitalizations,^22^ which may be a less biased COVID-19 outbreak metric,^20^ and this gives us confidence in the results presented here. As this devastating pandemic continues to unfold in New York, an early epicenter of the outbreak in the United States, we assume the number of confirmed COVID-19 cases will likely shift in response to improved testing and reporting practices, and over time, this will enable the emergence of more accurate and useful data. For now, we use the latest data available to us.^16^

Similarly, our sample is drawn from the New York City area, which reduces the generalizability of our findings to a particular portion of people in the city and surrounding areas. Furthermore, to reduce virus transmission and keep case-fatality rates as low as possible, the New York State government instituted a number of physical distancing control efforts which, in addition to school closures, included closures of nonessential businesses, cancellation of large-scale public gatherings, and restrictions on movement such as stay-at-home orders.^35^ In the present analysis, we used K-12 school closure dates alone to account for variation in time of enactment of these physical distancing measures across counties. While our inclusion of 1 variable is by no means comprehensive, we opted to use school closure dates because this measure was introduced early on in the initial response to the COVID-19 outbreak.^17,18^ However, if school closures were the only physical distancing measure enacted, intervention effectiveness would be substantially reduced and variation in COVID-19 incidence across counties would be magnified.^22,23^

We were unable to confirm whether individuals who tested positive for SARS-CoV-2 also lived in the area where testing was performed. However, local government agencies directed people to contact their local health department for COVID-19-related concerns,^36^ and since other physical distancing measures were being enacted around the same time as school closures,^17,18,35^ we are confident in the results reported here. Related, it is worth noting that the governor of New York issued a mandate on April 15, 2020, that required all people in the state to wear a face mask/covering when out in public and when using mass transit.^35^ This is a significant point because face masks/coverings may result in a large reduction in infection risk.^37^

Taken together, the dire circumstances in which we find ourselves demand better understanding of how distinctive geographic spaces influence infection incidence in order to potentially isolate the community-level factors that associate with a higher likelihood of COVID-19 diagnosis and disease progression. Scientists interested in disentangling which community factors relate to SARS-CoV-2 transmission may use our findings to better understand the connections between specific population subgroups and infectious disease dynamics. Decision makers, in turn, are obligated to incorporate empirical findings to guide public policy initiatives aimed at identifying and assisting at-risk populations.

## Supplementary Material

Supplemental data
