# Supplementary material for: County-Level Factors That Influenced the Trajectory of COVID-19 Incidence in the New York City Area
Source: Health Secur. 2021 Jun 17;19(Suppl 1):S-27–33. doi: 10.1089/hs.2020.0236 (PMC8236558; doi:10.1089/hs.2020.0236)
Supplement: Supplemental data [file Supp_TableS1.docx]

Supplemental Table 1. Means and Standard Deviations for Dependent and Independent Variables in 7 New York Counties (N = 327,578)

|  |  | *Variables* | *Bronx County* | | *Kings County* | | *Nassau County* | | *New York County* | | *Queens County* | | *Suffolk County* | | *Westchester County* | | *Diff^a^* |
| --- | --- | --- | --- | --- | --- | --- | --- | --- | --- | --- | --- | --- | --- | --- | --- | --- | --- |
| *Independent Variables* | | | Mean | SD | Mean | SD | Mean | SD | Mean | SD | Mean | SD | Mean | SD | Mean | SD |  |
|  | Time^b^ | |  |  |  |  |  |  |  |  |  |  |  |  |  |  |  |
|  |  | Time | 67.50 | 38.83 | 69.00 | 39.70 | 69.00 | 39.70 | 70.50 | 40.56 | 68.00 | 39.12 | 67.50 | 38.83 | 70.00 | 40.27 | <.001 |
|  |  | Time^2^ | 6052.50 | 5410.95 | 6325.00 | 5654.62 | 6325.00 | 5654.62 | 6603.50 | 5903.66 | 6142.67 | 5491.58 | 6052.50 | 5410.95 | 6510.00 | 5820.06 | <.001 |
|  |  | Time^3^ | 610538 | 692557 | 652257 | 739875 | 652257 | 739875 | 695835 | 789300 | 624240 | 708098 | 610538 | 692557 | 681100 | 772588 | <.001 |
|  | Physical Distancing | | |  |  |  |  |  |  |  |  |  |  |  |  |  |  |
|  |  | # Days from 1st Case to School Closure | 9 | -- | 12 | -- | 12 | -- | 15 | -- | 10 | -- | 9 | -- | 14 | -- | <.001 |
|  | Spatial Lag | | 26279.52 | 4124 | 37765.88 | 4639 | 35981.17 | 4174 | 42382.18 | 2453 | 36492.48 | 5563 | 37699.63 | 3690 | 31787.14 | 3125 | <.001 |
|  | Median Age | | 33.90 | <0.01 | 35.10 | <0.01 | 41.60 | <0.01 | 37.30 | <0.01 | 38.70 | <0.01 | 41.30 | <0.01 | 40.80 | <0.01 | <.001 |
|  | Population Total | | 1418207 | <0.01 | 2559903 | <0.01 | 1356924 | <0.01 | 1628706 | <0.01 | 2253858 | <0.01 | 1476601 | <0.01 | 967506 | <0.01 | <.001 |
|  | Concentrated Disadvantage (SD) | | 2.09 | -- | 0.66 | -- | -1.00 | -- | -0.32 | -- | 0.10 | -- | -0.93 | -- | -0.54 | -- | <.001 |
|  |  | % Adults <12 Years Education | 14.20 | <0.01 | 9.20 | <0.01 | 4.60 | <0.01 | 7.00 | <0.01 | 10.20 | <0.01 | 4.50 | <0.01 | 6.40 | <0.01 | <.001 |
|  |  | % Black Residents | 36.40 | <0.01 | 33.90 | <0.01 | 13.10 | <0.01 | 17.10 | <0.01 | 19.70 | <0.01 | 8.90 | <0.01 | 16.20 | <0.01 | <.001 |
|  |  | % Below Poverty Line | 26.10 | <0.01 | 17.10 | <0.01 | 4.00 | <0.01 | 12.70 | <0.01 | 10.50 | <0.01 | 4.70 | <0.01 | 6.20 | <0.01 | <.001 |
|  |  | % Receiving Public Assistance | 7.90 | <0.01 | 4.80 | <0.01 | 1.30 | <0.01 | 2.80 | <0.01 | 3.20 | <0.01 | 1.80 | <0.01 | 1.80 | <0.01 | <.001 |
|  |  | % Female Headed- Households | 29.90 | <0.01 | 18.20 | <0.01 | 11.20 | <0.01 | 11.10 | <0.01 | 16.00 | <0.01 | 11.10 | <0.01 | 12.50 | <0.01 | <.001 |
|  |  | % Unemployed | 6.30 | <0.01 | 4.40 | <0.01 | 2.80 | <0.01 | 3.80 | <0.01 | 4.00 | <0.01 | 3.10 | <0.01 | 3.80 | <0.01 | <.001 |
|  | 1st Case Date | | 3.08.2020 | | 3.05.2020 | | 3.05.2020 | | 3.02.2020 | | 3.07.2020 | | 3.08.2020 | | 3.03.2020 | | <.001 |
| *Dependent Variable* | | | |  |  |  |  |  |  |  |  |  |  |  |  |  |  |
|  | Number of Confirmed COVID-19 Cases | | 48,769 | | 61,432 | | 42,622 | | 29,731 | | 67,007 | | 42,466 | | 35,515 | | <.001 |
| Data are from March 2 through July 19, 2020, and are drawn from USAFacts,^16^ The City of New York website,^5^ Suffolk County Government,^18^ and the United States Census Bureau.^9^ | | | | | | | | | | | | | | | | | |
| ^a^ Significant difference (*P* value) was evaluated using 1-way multivariate analysis of variance (MANOVA) with county-level factors as the dependent variables and county as the independent variable. | | | | | | | | | | | | | | | | | |
| ^b^ Time is measured as the number of days since the first diagnosed case per county. Prior reports^20^, as well as our exploratory analyses, indicate that time is most appropriately captured by a cubic time function due to non-linearity. | | | | | | | | | | | | | | | | | |
|  |  |  |  |  |  |  |  |  |  |  |  |  |  |  |  |  |  |
| Abbreviation: SD, standard deviation. | | | | |  |  |  |  |  |  |  |  |  |  |  |  |  |
